# Neonatal Brain Structure, Early Life Context, and Academic Achievement among Children Born Very Preterm

**DOI:** 10.1016/j.jpedcp.2026.200230

**Published:** 2026-07-09

**Authors:** Stina Oftedal, Samudragupta Bora, Kerstin Pannek, Simona Fiori, Andrea Guzzetta, Alex M. Pagnozzi, Robert S. Ware, Paul B. Colditz, Roslyn N. Boyd, Joanne M. George

**Affiliations:** 1 Child Health Research Centre, Medicine and Behavioral Sciences, The University of Queensland, Centre for Children's Health Research, South Brisbane, QLD, Australia; 2 Queensland Cerebral Palsy and Rehabilitation Research Centre, Child Health Research Centre, Faculty of Health, Medicine and Behavioral Sciences, The University of Queensland, Centre for Children's Health Research, South Brisbane, QLD, Australia; 3 Health Services Research Center, University Hospitals Research & Education Institute, Department of Pediatrics, University Hospitals Rainbow Babies & Children's Hospital, Case Western Reserve University School of Medicine, Cleveland, OH; 4 Mater Research Institute, Faculty of Health, Medicine and Behavioral Sciences, The University of Queensland, Brisbane, Australia; 5 The Australian e-Health Research Centre, CSIRO, Herston, QLD, Australia; 6 School of Electrical Engineering and Computer Science, The University of Queensland, St Lucia, QLD, Australia; 7 Neuroscience and Medical Genetics Department, Meyer Children's Hospital, Florence, Italy; 8 Department of Neuroscience, Psychology, Pharmacology and Children's Health (NEUROFARBA), University of Florence, Florence, Italy; 9 Department of Clinical and Experimental Medicine, University of Pisa, Pisa, Italy; 10 IRCCS Stella Maris Foundation, Pisa, Italy; 11 Griffith Biostatistics Unit, Griffith University, Nathan Campus, Nathan, QLD, Australia; 12 Wesley Research Institute, Level 8, East Wing, Wesley Hospital, Auchenflower, QLD, Australia; 13 University of Queensland Centre for Clinical Research, Faculty of Health, Medicine and Behavioral Sciences, The University of Queensland, Royal Brisbane and Women's Hospital, Herston, QLD, Australia; 14 Perinatal Research Centre, Royal Brisbane and Women's Hospital, Herston, QLD, Australia; 15 Physiotherapy Department, Queensland Children's Hospital, Children's Health Queensland Hospital and Health Service, South Brisbane, QLD, Australia

**Keywords:** Early childhood, educational achievement, magnetic resonance imaging, neurodevelopment, preterm birth

## Abstract

**Objective:**

To examine whether neonatal brain abnormality severity was independently associated with academic achievement at 6 years and whether it modified the association between early life socioeconomic context and achievement in children born very preterm.

**Study design:**

A prospective cohort of 133 Australian infants born at <31 weeks’ gestation at a tertiary hospital was followed up at age 6 years. Early life socioeconomic context was assessed using a composite index across 6 variables at term-equivalent age (score range, 0-12) and dichotomized, with scores of 2 or higher classified as indicating higher disadvantage (n = 68 [51%]). Brain magnetic resonance imaging was performed early (at 32 weeks postmenstrual age) and at term-equivalent age and scored using the Kidokoro criteria (Global Brain Abnormality Score). Academic achievement was assessed using Woodcock-Johnson-IV Tests of Achievement, broad reading and mathematics clusters.

**Results:**

Higher disadvantage was consistently associated with poorer academic achievement outcomes (range b = −10.73 to −7.95; 95% CI, −16.90 to −2.30). Greater brain abnormality on early magnetic resonance imaging was associated with poorer mathematics (b = −0.95; 95% CI, −1.58 to −0.32) but not reading achievement. Greater brain abnormality at term-equivalent age was associated with poorer achievement in both reading (b = −0.86; 95% CI, −1.64 to −0.09) and mathematics (b = −1.00; 95% CI, −1.56 to −0.43). No statistically significant interactions were observed.

**Conclusions:**

Greater neonatal brain abnormality and higher early life disadvantage were independently associated with poorer academic achievement in children born very preterm. Brain abnormality severity did not modify the association between early life disadvantage and academic achievement. These findings support the assessment of both biological risk and socioeconomic disadvantage in neonatal follow-up.

**Clinical trial registry:**

Australian New Zealand Clinical Trials Registry ANZCTR12619000155190.

Preterm birth (<37 weeks’ gestation) is consistently linked to poorer academic outcomes, particularly in reading and mathematics.^1^, ^2^, ^3^ Children born preterm face greater odds of requiring special education services and grade repetition during their schooling.^4^^,^^5^ Educational vulnerabilities extend into adulthood, with evidence of lower economic and educational achievement persisting at least into their late 20s.^6^ Notably, these disparities increase with decreasing gestational age at birth.^6^ Higher social disadvantage is also an established risk factor for poor educational outcomes globally,^7^ and higher maternal social disadvantage is associated with an increased risk of preterm birth.^8^ Social disadvantage may amplify the adverse effects of preterm birth on educational attainment.^9^^,^^10^

Neonatal brain abnormality in very preterm infants (born at <32 weeks postmenstrual age) predicts later cognitive and academic outcomes, with the nature and timing of the injury moderating the strength of the associations.^11^, ^12^, ^13^, ^14^ Evidence further suggests that less favorable social circumstances and brain abnormality severity may interact to influence neurodevelopment. For example, in a prospective Canadian study of 126 very preterm neonates (born at 24-32 weeks' gestation), neonatal hippocampal development was associated with 18-month cognitive outcomes (Bayley Scales of Infant and Toddler Development III) only among children whose mothers had completed high school or less (n = 24), but not among those whose mothers had a college or postgraduate education (n = 108).^15^ In a Canadian cohort of 197 very preterm neonates (born at 24-32 weeks' gestation), brain injury was associated with lower cognitive scores at 4.5 years of age as measured using the Wechsler Primary and Preschool Scale of Intelligence (IV) only for the neonates whose mothers had an undergraduate or primary/secondary education (n = 163), whereas no difference was observed among children with and without brain injury for whose mothers held postgraduate degrees (n = 34).^16^ More recently, the findings from the large ELGAN cohort suggest that early white matter injury moderates the association between social disadvantage and cognitive trajectories from infancy to adolescence.^17^ In the ELGAN study, the apparent benefit of no social disadvantage was only evident in children without white matter injury, whereas the adverse effect of white matter injury was present, irrespective of social risk.^17^ This finding suggests that the presence of white matter injury diminishes the potential protective influence of the absence of social disadvantage. However, existing studies have largely relied on dichotomous indicators of brain injury and focused on cognitive outcomes measured in early or middle childhood. The use of a continuous measure of early brain abnormality may provide additional insights into how varying severity of brain injury relates to later academic achievement.

Thus, the present study aimed to determine if (1) academic achievement at 6 years of age differed by early life context and (2) neonatal brain abnormality severity was independently associated with academic achievement at 6 years in children born very preterm. We further explored whether brain abnormality severity modified the association between early life disadvantage and academic achievement, given prior evidence of effect modification using dichotomous indicators of brain injury.

## Methods

### Study Design and Sample

The PREBO-6 (Prediction of childhood Brain Outcomes in infants using neonatal MRI and concurrent clinical biomarkers) birth cohort recruited infants born before 31 weeks' gestation at the Royal Brisbane and Women's Hospital (Brisbane, Australia) between January 2013 and December 2019.^18^ Participants underwent 2 neonatal magnetic resonance imaging (MRI) scans, and those who were reassessed at 6 years of age were part of the PREBO-6 study (ANZCTR12619000155190).^18^ Infants with major chromosomal or congenital anomalies were excluded. Ethical approval was obtained from the Human Research Ethics Committees at the Royal Brisbane & Women's Hospital (HREC/12/QRBW/245), The University of Queensland (January 2013 [2012001060] and March 2019 [2019000426]) and the Children's Health Queensland Hospital and Health Service (February 2019 [HREC/19/QCHQ/49800]).

### Early Life Context

Early life socioeconomic context was assessed at term-equivalent age (TEA) using a parent-reported questionnaire that examined 6 areas, each scored from 0 to 2 (maximum score, 12), including family structure (0 = 2 caregivers [nuclear]; 1 = separated parents with dual custody, or cared for by other intact family; 2 = single caregiver), maternal age (0 = >21 years; 1 = 18-21 years; 2 = <18 years), language spoken at home (0 = English only; 1 = some English; 2 = no English), education level of primary caregiver (0 = tertiary educated; 1 = 11-12 years of formal schooling; 2 = <11 years of formal schooling), occupation type (0 = skilled/professional; 1 = semiskilled; 2 = unskilled), and employment status of the primary income earner (0 = full-time employment; 1 = part-time employment; 2 = unemployed/pension). The total score was dichotomized, with scores of 2 or more points classified as indicating higher early life disadvantage, that is, a high score of 2 points in 1 area or a moderate score of 1 point in 2 areas. This questionnaire has previously been used in Australian studies of very preterm infants.^19^ The cut-off point was informed by other similar scales used in cohorts of very preterm infants,^20^^,^^21^ recognizing that other studies use indices that capture more severe or structural indicators of social deprivation, such as public health insurance use or receipt of food assistance (eg, a score of 1 classified as high social risk).^17^ In contrast, our measure captures relative cumulative disadvantage within this cohort, distinguishing children exposed to multiple concurrent social risks from those with only 1 or no early life risk factors.

### Neonatal Brain Outcomes

Infants underwent early MRI at 32 weeks postmenstrual age, followed by a TEA MRI at 40 weeks postmenstrual age. Imaging was performed using a 3 Tesla Siemens Tim Trio (n = 60) or Skyra scanner (n = 68) (Erlangen, Germany), with infants placed in an incubator equipped with an MR-compatible neonatal head coil (LMT Lammers Medical Technology, Lübeck, Germany). To reduce scanner noise exposure, Natus Mini Muffs (Natus Medical Inc, San Carlos, CA) were used. No sedation was administered during scanning. MRI acquisition protocols for both cohorts have been detailed previously.^22^

All scans were independently rated by a neurologist trained in radiology and experienced in neonatal MRI interpretation, masked to clinical details apart from postmenstrual age at scan. Brain abnormality scores were derived using a modified version of the Kidokoro scoring system,^23^ as previously described,^24^ incorporating cystic lesions, signal changes, maturation, and volume loss. Raw regional measures (biparietal width, transcerebellar diameter, interhemispheric distance, ventricular diameter, deep gray matter area, and corpus callosum) were adjusted for postmenstrual age at scan^25^ and harmonized between imaging protocols.^26^ Subscale scores were calculated for cerebral white matter (range, 0-15), cortical gray matter (range, 0-8), deep gray matter (range, 0-6), and cerebellum (range, 0-6). Of note, 1 item (myelination delay) was always scored as 2 points at early MRI because myelination is not yet expected to be observable on MRI at this age. A global brain abnormality score (GBAS; range, 0-35) was computed by summing the subscale scores, with higher values indicating greater abnormality.^23^

### Academic Achievement

At 6 years of age, corrected for prematurity, children's academic achievement in reading and mathematics was assessed using the Woodcock-Johnson IV Tests of Achievement (WJ-IV, Australian version), administered by trained psychologists masked to the children's clinical history, social disadvantage, and MRI findings.^27^ The broad reading domain included letter-word identification, passage comprehension, and sentence reading fluency subtests. The broad mathematics domain comprised applied problems, calculation, and math facts fluency subtests.^27^ The WJ-IV broad reading and mathematics clusters demonstrate strong psychometric properties, with median reliability coefficients exceeding 0.90 and robust validity evidence for measuring academic achievement in school-aged children.^28^ Available evidence has shown that children with lower numeric competence measured at the start of formal schooling using the WJ applied problems subtest were enrolled in lower math track classes in high school and were less likely to enroll in college.^29^

### Statistical Analyses

Summary statistics are reported as mean ± SD or median (IQR) for continuous data and as frequency and percentage for categorical data. Geometric means were reported for Kidokoro scores because they were significantly skewed toward lower scores. Clinical birth and medical history characteristics were described for participants who were, and were not, in the 6-year follow-up group and for those with lower vs higher early life disadvantage. Participants with missing early life context or academic achievement data were excluded from the analyses.

Mixed-effects linear regression models with robust standard errors were used with “family” included as a random intercept to account for possible sibling nonindependence. Separate models were fitted for each outcome and MRI timepoint. Sex was included in all models as a covariate. Gestational age and neonatal morbidities were not included as covariates, because the GBAS reflects the downstream effects of prematurity and adjustment would risk overcontrolling for the pathway of interest. Assumptions of linear regression were assessed using standard diagnostic procedures.

For each outcome and time point, we estimated (1) the main effects models, including early life context and GBAS, and then fitted (2) a formal interaction model, including the main effects of both early life context disadvantage and GBAS, plus the interaction between the two. In addition to the primary main effects and interaction models, we conducted a secondary exploratory analysis to examine whether the association between early life context and academic outcomes differed across levels of neonatal brain abnormality using a conditional interaction model. This exploratory model (3) included the main effect of early life disadvantage plus the interaction between disadvantage and the GBAS, but did not include the GBAS as a main effect. The aim of this analysis was not to estimate the independent contribution of the GBAS, but to assess whether early life disadvantage had a differential association with outcomes depending on underlying neurobiological vulnerability, focusing on effect modification rather than total or independent effects.

Interaction terms were specified on the multiplicative scale, and coefficients represent the extent that associations vary as a function of the interacting variable. Negative values indicate attenuation of the association whereas positive values indicate amplification. Margin plots were generated for GBASs. Sensitivity analyses were conducted, restricting analyses to only those participants with MRIs at both timepoints and rerunning analyses after changing the myelination score at early MRI to zero instead of the default 2 points. Assumptions of linear regression were assessed using appropriate diagnostic plots and statistics. Statistical analyses were performed using Stata statistical software version 17.0 (StataCorp LCC, College Station, TX).

## Results

Of the 244 children eligible for inclusion in the current analyses, 156 (65%) consented to the neurodevelopmental follow-up component of the PREBO-6 study (Supplementary Figure 1; available at www.jpeds.com). Of these, a total of 133 children (74 males, aged 6.1 ± 0.1 years) from 119 families (n = 21 twin births) completed the WJ-IV assessment and had data available for analyses. Fifty-eight of 119 families (49%), representing 68 children, were classified as having “higher early life disadvantage” (Table I).

**Table I:** Birth and medical history characteristics of sample

Characteristics	Lower early life disadvantage (n = 65 children from 61 families)	Higher early life disadvantage (n = 68 children from 58 families)
Weeks' gestation at birth	28.9 [27^1/7^-30^1/7^]	28.0 [26^5/7^- 29^4/7^]
Birth weight (g)	1133 ± 297	1066 ± 315
Small for gestational age	3 (5)	3 (4)
Male sex	37 (57)	37 (54)
Multiple births	10 (15)	25 (37)
Completed course of antenatal corticosteroids	46 (71)	51 (75)
Postnatal corticosteroids	10 (15)	12 (18)
BPD∗	24 (37)	29 (43)
Patent ductus arteriosus	27 (42)	26 (38)
NEC diagnosed/suspected	3 (5)	3 (4)
Confirmed sepsis	2 (3)	2 (3)
Hydrocephalus	2 (3)	4 (6)
IVH grade 1 or 2	20 (31)	26 (38)
IVH grade 3 or 4	4 (6)	10 (15)
Periventricular leukomalacia	2 (3)	3 (4)
PMA at early MRI	32.0 [31.3-33.3]	32.1 [31.3-33.7]
PMA at TEA MRI	40.7 [40.3-41.6]	40.6 [40.1-41.3]
Weight (g) at early MRI	1478 [1274-1650]	1578 [1252-1739]
Weight (g) at TEA MRI	3010 [2722-3340]	3060 [2770-3400]
Early MRI GBAS†	4.8 ( × /1.6)	5.4 ( × /1.8)
Term MRI GBAS	2.8 ( × /2.1)	3.9 ( × /2.3)

*CA*, corrected age; *early MRI*, MRI acquired at 30-32 weeks postmenstrual age; *IVH*, intraventricular hemorrhage; *NEC*, necrotizing enterocolitis; *PMA*, postmenstrual age.

Data presented as number (%), median [IQR], mean ± SD, or geometric mean ( × /GSD).

∗ Requiring any respiratory support or supplemental oxygen for a chronic pulmonary disorder at 36 weeks.

† At early MRI, myelination is scored 2 points by default to reflect the lack of myelination, which is an expected finding at this early age, whereas at Term MRI it is scored: 0 = normal/age appropriate, 1 = mildly delayed, 2 = moderately to severely delayed.

Compared with children included in the current analyses, participants who attended the 6-year follow-up assessment but who did not complete a WJ-IV assessment (n = 23) had a greater proportion of multiple births (n = 13 [57%] vs n = 35 [26%]) and a greater proportion of children with bronchopulmonary dysplasia (BPD) (n = 15 [75%] vs n = 53 [40%]) (Supplementary Table 1; available at www.jpeds.com). There were no differences in clinical characteristics between those who attended vs those who did not attend follow-up or between the higher and lower disadvantage group, except that there were more multiple births in the higher (n = 24 [37%]) vs lower (n = 10 [15%]) disadvantage group (Supplementary Table 1; available at www.jpeds.com). There were no differences between the higher and lower disadvantage groups for Kidokoro subscores or GBAS on early MRI, but the higher disadvantage group had a higher GBAS at TEA MRI. The higher disadvantage group scored lower on both the broad reading and broad mathematics domains (Supplementary Table 2; available at www.jpeds.com).

In univariate models, higher disadvantage and greater brain abnormality at both early and TEA were each independently associated with poorer reading and mathematics achievement at age 6 years (Supplementary Table 3; available at www.jpeds.com). In multivariate models including both exposures, early life disadvantage showed a consistent, independent association with lower reading and mathematics achievement across MRI timepoints (early MRI reading: b = −10.57; 95% CI, −16.69 to −4.45; mathematics: b = −7.95; 95% CI, −13.60 to −2.30; and TEA MRI reading: b = −10.73; 95% CI, −16.90 to −4.56; mathematics: b = −8.58; 95% CI, −14.19 to −2.96) (Table II). Greater brain abnormality (GBAS) was also independently associated with poorer academic outcomes in most multivariate models (early MRI mathematics b = −0.95; 95% CI, −1.58 to −0.32; TEA-MRI reading: b = −0.84; 95% CI, −1.60 to −0.07; mathematics : b = −0.97; 95% CI, −1.52 to −0.42), but not early MRI and reading (b = −0.84; 95% CI, −1.70 to 0.01) (Table II). However, in the primary models including main effects and interaction terms, no statistically significant interactions were detected between early life disadvantage and brain abnormality scores for either reading or mathematics outcomes, and estimates were imprecise with wide CIs (Table II), providing no clear evidence of effect modification.

**Table II:** Associations between early life disadvantage, GBASs, and academic achievement at 6 years of age

MRIs	Main effects model	Interaction model	Conditional interaction model	Main effects model	Interaction model	Conditional interaction model
	b [95% CI]	b [95% CI]	b [95% CI]	b [95% CI]	b [95% CI]	b [95% CI]
	WJ-IV Broad Reading Achievement at 6 years old			WJ-IV Broad Mathematics Achievement at 6 years old		
Early MRI						
Lower early life disadvantage	Ref	Ref	Ref	Ref	Ref	Ref
Higher early life disadvantage	−10.57	−6.97	−6.97	−7.95	−7.82	−7.82
	[−16.69 to −4.45]	[−19.22 to 5.28]	[−19.22 to 5.28]	[−13.60 to −2.30]	[−17.86 to 2.22]	[−17.86 to 2.22]
Early MRI GBAS	−0.84	−0.44	n/a	−0.95	−0.94	n/a
	[−1.70 to 0.01]	[−2.50 to 1.59]		[−1.58 to −0.32]	[−2.16 to 0.29]	
Lower early life disadvantage × Early MRI GBAS	n/a	Ref	−0.44	n/a	Ref	−0.94
			[−2.42 to 1.54]			[−2.16 to 0.29]
Higher early life disadvantage × Early MRI GBAS	n/a	−0.62	−1.07	n/a	−0.02	−0.96
		[−2.69 to 1.44]	[−1.73 to −0.40]		[−1.42 to 1.38]	[−1.66 to −0.26]
TEA MRI						
Lower early life disadvantage	Ref	Ref	Ref	Ref	Ref	Ref
Higher early life disadvantage	−10.73	−11.35	−11.35∗∗	−8.58	−9.56	−9.56
	[−16.90 to −4.56]	[−20.11 to −2.59]	[−20.11 to −2.59]	[−14.19 to −2.96]	[−17.50 to −1.63]	[−17.50 to −1.63]
TEA MRI GBAS	−0.84	−0.94	n/a	−0.97	−1.13	n/a
	[−1.60 to −0.07]	[−2.62 to 0.75]		[−1.52 to −0.42]	[−2.05 to −0.21]	
Lower early life disadvantage × TEA MRI GBAS	n/a	Ref	−0.94	n/a	Ref	−1.13
			[−2.62 to 0.75]			[−2.05 to −0.21]
Higher early life disadvantage × TEA MRI GBAS	n/a	0.16	−0.78	n/a	0.26	−0.87
		[−1.65 to 1.97]	[−1.47 to −0.09]		[−0.90 to 1.41]	[−1.56 to −0.18]

*Early MRI*, MRI acquired at 30-32 weeks postmenstrual age.

Mixed multiple linear effects models, all adjusted for participant sex and multiple births (twins).

In the conditional interaction models examining early MRI, the main effect of early life disadvantage was nonsignificant (reading: b = −6.97; 95% CI, −19.22 to 5.28; mathematics: −7.82; 95% CI, −17.86 to 2.22). Greater brain abnormality was negatively associated with reading and mathematics achievement among children with higher disadvantage (reading: b = −1.07; 95% CI, −1.73 to −0.40; mathematics: b = −0.96; 95% CI, −1.66 to −0.26), whereas corresponding associations were not statistically significant among children with lower disadvantage (Table II).

In conditional models using TEA MRI, higher disadvantage showed a significant negative main effect on both reading (b = −11.35; 95% CI, −20.11 to −2.59) and mathematics achievement (b = −9.56; 95% CI, −17.50 to −1.63). Greater TEA brain abnormality was associated with lower reading achievement among children with higher disadvantage (b = −0.78; 95% CI, −1.47 to −0.09), but not lower disadvantage (Table II). For mathematics outcomes, greater TEA brain abnormality was significantly associated with poorer achievement in both higher (b = −1.13; 95% CI, −2.05 to −0.21) and lower (b = −0.87; 95% CI, −1.56 to −0.18) disadvantage groups (Table II). Findings from conditional models should be interpreted descriptively, as formal tests of interaction in primary models did not provide evidence of a statistically significant effect modification.

Margins plots illustrating the associations between brain abnormality scores and academic outcomes within early life disadvantage strata are presented in Figure. There were no substantive differences in the regression results when the sample was restricted to those with MRI completed at both early and TEA timepoints (n = 126). As expected, changing the myelination score to zero at early MRI did not affect the estimated associations between GBAS and academic outcomes (ie, regression coefficients, CIs), because the relative differences between individuals were preserved. The model intercept decreased accordingly, reflecting rescaling of the predictor.

**Figure:**
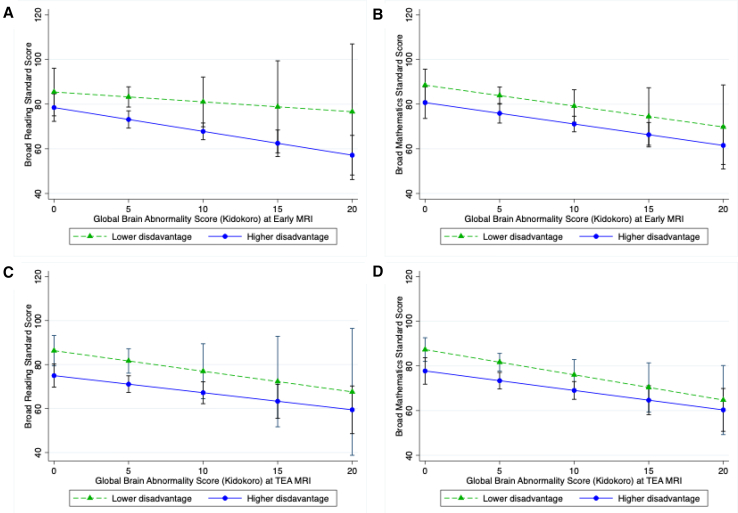
**A-D,** Margins plots of Woodcock Johnson-IV Reading and Mathematics Achievement vs Kidokoro GBAS at early (32 weeks PMA) and TEA (40 weeks PMA) MRI by early life disadvantage group, regression adjusted for sex and multiple births (twins) and with interaction term (Early life disadvantage × GBAS) included. *PMA*, postmenstrual age.

## Discussion

In this contemporary prospective cohort of children born very preterm, both greater brain abnormality severity and higher early life disadvantage were independently associated with poorer academic achievement at 6 years of age. Early life disadvantage showed a consistent and substantial association with both reading and mathematics outcomes, while greater brain abnormality was associated with poorer academic achievement in most models. These findings demonstrate the importance of considering both biological and social vulnerabilities when evaluating early academic risk in very preterm populations.

Associations between brain abnormality and reading achievement differed between early and TEA MRI timepoints. Brain abnormality severity at TEA showed a more consistent association with reading achievement across early life contexts, whereas early brain abnormality was associated with poorer reading achievement primarily among children experiencing higher disadvantage. Differences may reflect changes in brain abnormality between MRI timepoints (Table I and Supplementary Table 2; available at www.jpeds.com), as well as methodological considerations. One item (myelination delay), which has traditionally been scored 2 at early MRI to reflect a lack of myelination (and considered normal at this early age),^24^ is frequently scored 0 at TEA. Therefore, observed changes in the score itself that result from this scoring item may not imply true biological change. As a result, GBASs at early and term MRI are not directly comparable in absolute terms; however, this represents a constant offset at the early timepoint and does not affect associations with outcomes, which depend on relative differences between individuals. Further work is needed to determine whether the changes observed on MRI from early to TEA can meaningfully add to our understanding of expected academic outcomes. In addition, limited numbers of children with more severe abnormalities, particularly in the lower early life disadvantage group, resulted in wider CIs at the higher score ranges, which limits precision.

Formal tests of interactions between brain abnormality severity and early life disadvantage were not statistically significant. Exploratory conditional models were used to examine associations between brain abnormality severity and academic outcomes within the social disadvantage groups. These analyses indicated that the association between brain abnormality severity and academic outcomes was similar in magnitude across disadvantage groups, but estimated more precisely in children in the higher social disadvantage group. CIs were wider for children with lower disadvantage, limiting statistical power in this group. The similarity of effect estimates across disadvantage groups, together with nonsignificant interaction terms in the formal interaction models, suggest no clear evidence of effect modification. However, the study may have been underpowered to detect interaction effects, particularly given the small number of children with severe brain abnormalities in the lower disadvantage group. Accordingly, the absence of a statistical interaction should not be interpreted as evidence of no underlying biological interaction.

Methodological differences make it challenging to make a direct comparison with previous prospective cohort studies examining interactions between early life context and neonatal brain injury. Prior Canadian studies reported that an association between brain injury and cognition at 18 months^15^ and 4.5 years^16^ was only present in mothers with lower levels of educational attainment, with little evidence of brain injury effects among children of more highly educated mothers. These studies examined early cognitive outcomes assessed in infancy or the preschool years. More recently, findings from the ELGAN cohort showed that early white matter injury moderated the association between social disadvantage and cognitive trajectories from infancy through to adolescence.^17^ Specifically, the apparent positive effect of no social disadvantage was only evident among children without white matter injury, whereas the adverse effect of white matter injury was evident irrespective of social risk.^17^ This pattern suggests that the presence of white matter injury diminishes the potential protective influence of having no social disadvantage. In contrast, the present study assessed brain abnormality using a continuous severity score and focused on academic achievement at 6 years of age. Although this approach captures graded variation in brain abnormalities, it may be less sensitive to the threshold effects associated with the presence of brain injury as defined in the previous literature, particularly in a smaller sample. Cohort differences may also play a role, as prior studies may have included infants with greater clinical severity (eg, higher rates of small for gestational age, sepsis, or BPD), potentially increasing variability and the likelihood of detecting associations. Measurement differences, sample characteristics, and limited power may explain the absence of statistically significant interaction effects in this study.

Our study has several strengths, including the prospective follow-up, separate analyses for early and TEA MRI, masked outcomes assessment, and the use of gold standard assessments of early academic achievement. Limitations include the limited statistical power for interaction analyses; selective noncompletion of assessment among children with multiple birth and BPD, which may have resulted in conservative estimates of academic risk as these children are at greater risk of both more severe brain abnormality and poorer academic outcomes; and parsimonious covariate adjustment necessitated by sample size and interaction modelling. Residual confounding cannot be excluded and findings should be interpreted as associative rather than causal.

The cohort was recruited in Australia, a setting with universal health care, publicly funded early intervention, and additional school-based support, which may limit generalizability to other contexts. Findings are also specific to children born very preterm and may not apply to those born at moderate to late preterm. The relatively high proportion of infants identified as having higher early life disadvantage than Australian population averages likely reflects the underlying socioeconomic profile of the population served by the tertiary hospital from which the cohort was recruited,^30^ combined with the well-established association between higher social disadvantage and preterm birth.^8^

This study shows that both higher early life disadvantage and greater brain abnormality are independently associated with poorer academic achievement at 6 years of age in children born very preterm, and differences in variable scaling (binary vs continuous) should be considered when comparing effect magnitudes. Children born very preterm with either higher early life disadvantage or greater neonatal brain abnormality represent groups that may benefit from closer educational surveillance and targeted early support. Given that preterm birth is more common among mothers experiencing social disadvantage, this compounded vulnerability emphasizes the need to integrate social factors into both hospital discharge planning and neurodevelopmental follow-up. Further research is needed to clarify the pathways linking early brain abnormalities and later academic achievement, including whether trajectories of brain maturation from early age to TEA provide additional insights into developmental vulnerability. Early MRI findings, when considered alongside indicators of social risk, may help to identify children who could benefit from closer monitoring and early support. Ensuring timely access to early support that is responsive to each child and family's unique circumstances could play an important role in promoting better long-term educational outcomes for children born very preterm.

## CRediT authorship contribution statement

**Stina Oftedal:** Writing – review & editing, Writing – original draft, Visualization, Methodology, Formal analysis, Conceptualization. **Samudragupta Bora:** Writing – review & editing, Supervision, Methodology, Investigation, Funding acquisition, Conceptualization. **Kerstin Pannek:** Writing – review & editing, Project administration, Investigation, Funding acquisition, Data curation. **Simona Fiori:** Writing – review & editing, Investigation. **Andrea Guzzetta:** Writing – review & editing, Investigation. **Alex M. Pagnozzi:** Writing – review & editing, Funding acquisition. **Robert S. Ware:** Writing – review & editing, Validation, Supervision, Methodology, Funding acquisition. **Paul B. Colditz:** Writing – review & editing, Project administration, Investigation, Funding acquisition. **Roslyn N. Boyd:** Writing – review & editing, Funding acquisition. **Joanne M. George:** Writing – review & editing, Supervision, Resources, Investigation, Funding acquisition, Data curation, Conceptualization.

## Declaration of Competing Interest

JG was supported by the 10.13039/501100022099Cerebral Palsy Alliance, the Financial 10.13039/100028927Markets 10.13039/100028927Foundation for Children, the 10.13039/501100000925National Health and Medical Research Council and the Mary McConnel Career Boost Program for Women in Pediatric Research. RB was supported by the 10.13039/501100000925National Health and Medical Research Council. AG was supported by the 10.13039/501100003196Italian Ministry of Health - Ricerca Corrente (2026). The funders were not involved in study design, collection, analysis or interpretation of data, writing of the report, nor the decision to submit the article for publication.

The authors report no known competing financial interests or personal relationships that could have appeared to influence the work reported in this paper.
