# Supplementary material for: Neonatal Brain Structure, Early Life Context, and Academic Achievement among Children Born Very Preterm
Source: J Pediatr Clin Pract. 2026 Jul 9;21:200230. doi: 10.1016/j.jpedcp.2026.200230 (PMC13463981; doi:10.1016/j.jpedcp.2026.200230)
Supplement: Supplementary file 1 [file mmc1.docx]

**Supplementary Figure 1: Study flow chart**

PREBO-6

n=243 eligible

n=211 families

PPREMO and PREBO

N=249 eligible children

N=217 families

PREBO-6

N=161 attended

Neuropsychology follow-up

PREBO-6

n=134 completed WJ-IV test of academic achievement

Broad reading=132

Broad maths=134

Reasons for not completing (n=22):

Child fatigue n=2

Behavioural difficulties: n=4

Refusal to participate: n=3

Parent declined: n=4

Time constraints: n=4

Other: n=4

Reasons for no Neuropsychology follow-up (n=82)

n=1 missed by study team

n=37 declined

n=44 lost to follow up

PREBO-6

Current analyses

Broad reading n=131

Broad maths n=133

Reasons for not included:

Missing social disadvantage variable = 1

Kidokoro score at Early MRI

n=133 children

n=119 families

Kidokoro score at Term-equivalent-MRI

n=126 children

n=114 families

Reasons for ineligibility (n=7)

New diagnosis of medical condition, n=1

Not yet turned 6 years corrected age, n=5
