# Supplementary material for: Neonatal Brain Structure, Early Life Context, and Academic Achievement among Children Born Very Preterm
Source: J Pediatr Clin Pract. 2026 Jul 9;21:200230. doi: 10.1016/j.jpedcp.2026.200230 (PMC13463981; doi:10.1016/j.jpedcp.2026.200230)
Supplement: Supplementary file 2 [file mmc2.docx]

| **Supplementary Table 1: Comparison of included and not included participants** | | | | |
| --- | --- | --- | --- | --- |
|  | **Included in analyses**  **n=133** | **Attended 6-year follow-up**  **(incomplete data) n=23^1^** | **Did not attend follow-up**  **n=88** | **p-value** |
| Gestational age at birth (weeks) | 28.4 (26.9-29.9) | 28.3 (26.6-30.4) | 28.9 (27.4-29.7) | 0.41 |
| Birth weight (g) | 1099 (307) | 1029 (310) | 1160 (298) | 0.13 |
| Small for gestational age (SGA) | 6 (5%) | 2 (9%) | 5 (6%) | 0.59 |
| Males | 74 (56%) | 11 (48%) | 46 (52%) | 0.74 |
| Multiple births | 35 (26%) | 13 (57%) | 24 (27%) | 0.017 |
| High early life disadvantage | 68 (51%) | 13 (59%) | 53 (61%) | 0.35 |
| Completed course of antenatal corticosteroids | 97 (73%) | 17 (74%) | 59 (67%) | 0.62 |
| Postnatal corticosteroids | 22 (17%) | 3 (15%) | 9 (11%) | 0.49 |
| Bronchopulmonary dysplasia | 53 (40%) | 15 (75%) | 32 (39%) | 0.010 |
| Patent ductus arteriosus | 53 (40%) | 6 (26%) | 29 (33%) | 0.36 |
| NEC diagnosed/ suspected | 6 (5%) | 0 (0%) | 5 (6%) | 0.72 |
| Confirmed sepsis | 4 (3%) | 3 (15%) | 5 (6%) | 0.060 |
| Hydrocephalus | 6 (5%) | 2 (10%) | 2 (2%) | 0.26 |
| IVH grade 1 or 2 | 46 (35%) | 7 (30%) | 19 (22%) | 0.11 |
| IVH grade 3 or 4 | 14 (11%) | 3 (13%) | 5 (6%) | 0.36 |
| Periventricular leukomalacia | 5 (4%) | 1 (5%) | 3 (4%) | 0.88 |
| PMA at Early MRI | 32.0 [31.3-33.6] | 32.4 [31.7-33.7] | 32.0 [31.4-32.9] | 0.22 |
| PMA at TEA MRI (6 missing) | 40.7 [40.1-41.6] | 41.1 [40.4-42.0] | 40.7 [40.0-41.4] | 0.18 |
| Weight (grams) at Early MRI | 1532 [1260-1705] | 1395 [1302-1677] | 1454 [1294-1652] | 0.66 |
| Weight (grams) at TEA MRI | 3060 [2730-3400] | 3230 [2920-3632] | 3000 [2700-3500] | 0.22 |
| Total Kidokoro score at Early MRI* | 5.0 [3.0-7.0] | 5.0 [3.0-9.0] | 5.0 [4.0-7.0] | 0.92 |
| Total Kidokoro score at TEA MRI* | 3.0 [1.0-5.0] | 3.0 [2.0-8.0] | 3.0 [2.0-5.5] | 0.33 |
| Data are presented as mean (SD) or median [IQR] for continuous measures, and No. (%) for categorical measures; p-value, One-Way ANOVA for continuous data and Pearson’s X^2^ test for categorical data; ^1^ Missing social disadvantage (n=1), Partially completed Woodcock-Johnson-4 assessment (n=7), Did not attempt Woodcock-Johnson-4 assessment (n=15).*At Early MRI, myelination is scored 2 points by default because myelination cannot be observed at this early age, whereas at Term MRI it is scored: 0 = normal/age appropriate, 1=mildly delayed, 2=moderately-to-severely delayed. | | | | |
