# Supplementary material for: Neonatal Brain Structure, Early Life Context, and Academic Achievement among Children Born Very Preterm
Source: J Pediatr Clin Pract. 2026 Jul 9;21:200230. doi: 10.1016/j.jpedcp.2026.200230 (PMC13463981; doi:10.1016/j.jpedcp.2026.200230)
Supplement: Supplementary file 3 [file mmc3.docx]

| **Supplementary Table 2: Neonatal neurological brain abnormality and academic achievement at 6 years old by early life disadvantage** | | | | |
| --- | --- | --- | --- | --- |
|  | | **Low early life disadvantage** | **High early life disadvantage** | **p-value** |
| **Early MRI** | | **n=65** | **n=68** |  |
| Kidokoro subscale scores | White matter | 3.6 (×/1.5)* | 2.1 (×/1.9)* | 0.30 |
|  | Cortical gray matter | 1.2 (×/1.4) | 1.5 (×/1.5) | 0.43 |
|  | Deep gray matter | 1.2 (×/1.4) | 1.4 (×/1.5) | 0.15 |
|  | Cerebellum | 1.5 (×/1.6) | 1.3 (×/1.6) | 0.44 |
|  | Global brain abnormality | 4.8 (×/1.6) | 2.8 (×/2.1) | 0.17 |
| **TEA MRI** | | **n=65** | **n=61** |  |
| Kidokoro subscale scores | White matter | 2.1 (×/1.9) | 2.5 (×/2.2) | 0.17 |
|  | Cortical gray matter | 1.5 (×/1.5) | 1.8 (×/1.4) | 0.23 |
|  | Deep gray matter | 1.4 (×/1.5) | 1.3 (×/1.6) | 0.89 |
|  | Cerebellum | 1.3 (×/1.6) | 1.5 (×/1.7) | 0.56 |
|  | Global brain abnormality | 2.8 (×/2.1) | 3.9 (×/2.3) | 0.031 |
| **Academic Outcomes at 6 Years on Woodcock-Johnson IV** | | **n=65** | **n=68** |  |
| Broad Reading Standard Score | | 79.0 (69.0-94.0) | 69.5 (61.0-79.0) | <0.001 |
| Broad Mathematics Standard Score | | 81.0 (74.0-96.0) | 71.0 (59.0-86.5) | 0.002 |
| One-Way ANOVA (p<0.05); Data presented as mean (standard deviation) or geometric mean (×/GSD); Early MRI: MRI acquired 30-32 weeks postmenstrual age; MRI: Magnetic Resonance Imaging; PMA: Postmenstrual Age; TEA: Term Equivalent Age; ). *At Early MRI, myelination is scored 2 points by default because myelination cannot be observed at this early age, whereas at Term MRI it is scored: 0 = normal/age appropriate, 1=mildly delayed, 2=moderately-to-severely delayed. | | | | |
