# Supplementary material for: Neonatal Brain Structure, Early Life Context, and Academic Achievement among Children Born Very Preterm
Source: J Pediatr Clin Pract. 2026 Jul 9;21:200230. doi: 10.1016/j.jpedcp.2026.200230 (PMC13463981; doi:10.1016/j.jpedcp.2026.200230)
Supplement: Supplementary file 4 [file mmc4.docx]

| **Supplementary Table 3: Univariate mixed regression models for independent variables** | | |
| --- | --- | --- |
|  | **Broad Reading** | **Broad Mathematics** |
|  | **b [95%CI]** | **b [95%CI]** |
| **Univariate analyses social disadvantage** | | |
| Low early life disadvantage | Ref | Ref |
| High early life disadvantage | -11.27 | -8.81 |
|  | [-17.37,-5.18] | [-14.45,-3.16] |
| **Univariate analyses Ealy-MRI Global Brain Abnormality Score (30-32 weeks PMA)** | | |
| Early MRI GBAS | -0.90 | -1.03 |
|  | [-1.76,-0.03] | [-1.66,-0.39] |
| **Univariate analyses TEA MRI Global Brain Abnormality Score (40-42 weeks PMA)** | | |
| TEA MRI GBAS | -0.93 | -1.10 |
|  | [-1.72,-0.14] | [-1.66,-0.54] |
| MRI: Magnetic Resonance Imaging; TEA: Term equivalent age. | | |
